# Composites of silica with immobilized cholinesterase incorporated into polymeric shell

**DOI:** 10.1186/s11671-015-0808-4

**Published:** 2015-02-27

**Authors:** Victoriya Payentko, Alexander Matkovsky, Yulia Matrunchik

**Affiliations:** Department of Amorphous and Structurally Ordered Oxides, Chuiko Institute of Surface Chemistry, National Academy of Sciences of Ukraine, 17, General Naumov str., Kyiv, 03164 Ukraine; Department of Composite Materials, The Institute of General and Inorganic Chemistry, National Academy of Sciences of Belarus, 9/1 Surganov Street, Minsk, BY-220072 Republic of Belarus

**Keywords:** Cholinesterase, Acetylcholinesterase, Polysiloxane matrix, Nanocomposite, Enzyme activity, 68.35.Ct, 81.07.Pr, 82.70Gg, 83.80.Hj

## Abstract

Synthetic approaches for new nanocomposite materials with relatively high cholinesterase activity have been developed. The peculiarity of the formation of such systems is the introduction of cholinesterase into polymer with subsequent incorporation on the ready-made silica particles and into the polysiloxane matrixes during sol-gel synthesis. Evaluation of the cholinesterase activity has been fulfilled through the imitation of the acetylcholine chloride decomposition reaction. Values of activity for cholinesterase nanocomposites demonstrated in this work are higher than those for the native cholinesterase. The higher activity of cholinesterase contained in nanocomposites was found for those prepared using highly dispersed silica.

## Background

Nanocomposites with immobilized bio-molecules are in focus of interest due to the reinforced stability of embedded bio-molecule in comparison with the initial and therefore prolonged catalytic activity. Such bio-specimens are widely used in medicine [1], biochemical analysis [2], etc. The application of immobilized biocatalysts [3] in analytics is very promising due to their high selectivity.

However, complications in the preparation procedure of purified (or partly purified) ferments have an effect on their value and as a result contract the sphere of their usage. The application of unpurified ferment specimens in biochemical analysis allows reducing the price of analytical assays.

Nanocomposites with high activity of immobilized cholinesterase (CHE) were synthesized using the liver homogenate of domestic hen *Gallus gallus* and acetylcholinesterase (ACHE) obtained from *Electroporus electricum* (electric eel) [4,5]; their stability towards the influence of medium [6] was achieved utilizing a method of double protection of the ferment by encapsulation in polymeric shell and further introduction in the SiO_2_ matrix.

The objective of this work is to identify the silica matrix influence on the ferment activity of the resulting nanocomposite. Two approaches of introducing the polymer-protected ferment into silica matrix were proposed: a) by performing the sol-gel synthesis by acidifying sodium metasilicate until pH = 6 (this pH ensures maintenance of the ferment activity) and b) by utilization of ready-made highly dispersed pyrogenic silica suspended in water. The content of SiO_2_ and values of pH were the same for all samples, but the samples differ in cholinesterases of different origin.

## Methods

### Materials

The materials are as follows: sodium metasilicate (Sigma-Aldrich, St. Louis, MO, USA), highly dispersed pyrogenic silica (A300, Kalush, Ukraine), ACHE solution from electric eel (mw = 280 kDa, Sigma-Aldich), polymeric composition on the base of poly(vinyl alcohol) (PVA) (JVP, Japan) and polyacrylic acid (PAA) (Sigma-Aldrich) (PAA:PVA = 10:1, pH = 6) synthesized in regard to [7], 0.067 M solution of Na, K-phosphate buffer (pH 6), 1 M solution of hydrochloric acid (Sigma-Aldrich), sodium chloride (Sigma-Aldrich), sodium hydroxide (Sigma-Aldrich), magnesium chloride hexahydrate (Sigma-Aldrich), acetylcholine chloride (Sigma-Aldrich), and CHE from the liver homogenate of *G. gallus* which was obtained as shown elsewhere [8].

### Assay of enzyme activity

**Figure 1:**
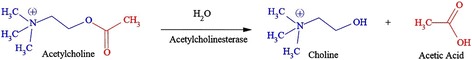
**Hydrolysis of acetylcholine using acetylcholinesterase.**

To evaluate the cholinesterase activity, a trimetric method [9] was employed according to Figure 1. Nanocomposite samples were placed in a water bath maintained at 38°C and the pH adjusted to 7.4 with 0.1 N NaOH; the acetylcholine was then added to the mixture.

The acetic acid that liberates from acetylcholine hydrolysis was neutralized and the pH maintained with NaOH by means of an automatic recording titrator (Titronic Easy, Darmstadt, Germany). A blank was determined first and analysis followed. Enzyme activity was calculated from the quantity of NaOH added per unit time and expressed as unit/mg of solid matter for composites and unit/mg of the enzyme solution for native ferment.

Synthesis of composite materials with immobilized cholinesterase:

1. The sol of SiO_2_ was obtained as described earlier [4] using sodium metasilicate and hydrated silicic acid diluted by water in proportion 1:2. The sol of SiO_2_ (10 ml) was acidified by 1 M HCl to pH 6, and then 10 ml of suspension of polymer (5.66 wt %) in phosphate buffer was added under vigorous stirring to the sol of SiO_2_. Samples were marked as sol-gel.
2. Highly dispersed pyrogenic silica A300 (0.25 g) was introduced to the liver homogenate/water suspension of the ferment content equal to the previous procedure with the maintenance of pH 6 and the same composition. Samples were marked with A300.

The obtained samples were dried out at 25°C tile air-dried state with further maintenance at 4°C.

The methods used were scanning electron microscopy (SEM) (JSM-5610 LV, JEOL Ltd., Akishima, Tokyo, Japan) and potentiometric titration (Titronic Easy, Germany).

## Results and discussion

The catalytic activities of cholinesterases of different origin (homogenate of the *G. gallus* liver and solution of ACHE from the electric eel) have been studied in native and immobilized states. The obtained results are presented in Table 1.

**Table 1:** **Activity of native and immobilized cholinesterases**

**Sample**	**Enzyme activity, unit** ^**a**^ **/mg**
Liver homogenate of the *Gallus gallus*	1.15 ± 0.04
ACHE from electric eel	1.88 ± 0.09
1. Sol-gel, liver homogenate of the *Gallus gallus*	1.27 ± 0.05
2. Sol-gel, ACHE from electric eel	2.15 ± 0.01
3. A300, homogenate of the liver of *Gallus gallus*	1.49 ± 0.01
4. A300, ACHE from electric eel	2.25 ± 0.03

^a^Unit definition: One unit will hydrolyze 1.0 μmol of acetylcholine to choline and acetate per minute at pH 8.0 at 37°C [9].

**Figure 2:**
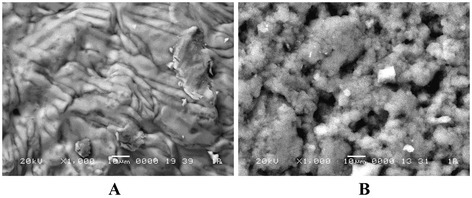
**Microphotographs of two types of hybrid materials obtained using scanning electron microscopy (SEM**)**.** Formation of silica components in composites **(A)** through the sol-gel process and **(B)** by means of A300 particle aggregation.

Enzyme specimen in immobilized state demonstrates higher values of catalytic activity compared to native forms (Table 1). Utilizing highly dispersed silica (A300) as silica component in composite allows retaining the higher values of catalytic activity in the reaction of acetylcholine chloride hydrolysis. This may be explained by strong difference in morphology of composites obtained by two procedures which may be seen from SEM microphotographs (Figure 2).

Figure 2A shows that the silica component formed through sol-gel method, although it has a complex surface relief, nevertheless is smoothed due to the uniform distribution of polymeric component in silica matrix. Whereas the silica component is formed by means of A300 particle aggregation, the high heterogeneity is observed due to diverse sizes of the obtained aggregates.

Such difference in morphology may facilitate the propagation of diffusion processes of delivery of the substrate to immobilized ferment. It is known [10] that hydrogen bonds between silica particles and water-soluble polymers are absent at pH 6. The formation of silica component takes place at the expense of interparticle interactions, and therefore, the interaction forces between the solid and dispersion medium have no significant influence. Herein [11], we have showed that in the silica-polymer system obtained at pH 6, after removal of the polymeric component by way of soft oxidation, the pore structure of the resulting silica matrix was insignificantly dependent on the nature of polymers. So, the functional structure of the obtained composites is represented by enzyme in the polymeric shell (which creates effect of the cell wall similar to *in vivo*) surrounded by silica matrix.

## Conclusions

It has been found that cholinesterases, immobilized into the organic-inorganic nanocomposite, have relatively higher enzyme activity in comparison with the native form. The considerable influence on the properties of immobilized enzyme has been attributed to the nature of silica’s component. In the case of the sol-gel, the resulting sample structure is more dense and uniform, whereas the highly dispersed silica provides a diverse heterogeneous structure that possibly facilitates the diffusion process which results in an increase in the enzyme activity.

## Abbreviations

CHE: cholinesterase
ACHE: acetylcholinesterase
PVA: poly(vinyl alcohol)
PAA: polyacrilic acid
SEM: scanning electron microscopy
